# Exploring quality of life among elderly persons living with HIV in Accra, Ghana

**DOI:** 10.1371/journal.pone.0324824

**Published:** 2025-06-13

**Authors:** Marijanatu Abdulai, Philip Teg-Nefaah Tabong, Harriet Affran Bonful, Adolphina Addo-Lartey, Bismark Sarfo

**Affiliations:** 1 Department of Epidemiology and Disease Control, School of Public Health, University of Ghana, Legon, Accra, Ghana; 2 National AIDS/STI Control Programme, Public Health Division of the Ghana Health Service, Legon, Accra, Ghana; 3 Department of Social and Behavioural Science, School of Public Health, University of Ghana, Legon, Accra, Ghana; University of the Witwatersrand Johannesburg, SOUTH AFRICA

## Abstract

**Introduction:**

The introduction and rapid scale-up of AntiRetroviral Treatment (ART) has contributed to a significant reduction in HIV-associated morbidities and mortalities, resulting in increasing life expectancy. Ageing with HIV presents several challenges which affect peoples’ well-being and quality of life (QoL). Despite this, there is scarcity of research on the QoL of Elderly Persons Living with HIV (EPLHIV) in Sub-Saharan Africa (SSA), particularly in Ghana. This study, therefore, examines the QoL of the elderly population using a qualitative approach.

**Methods:**

A purposive sampling method was used to select eight (8) participants for Key Informant Interviews (KIIs) and twenty-nine (29) participants for Focus Group Discussions (FGDs). In all, eight (8) KIIs with stakeholders and four (4) FGDs with EPLHIV were used to collect data for this study. Both the KIIs and FGDs interview guides addressed the four domains of the WHO’s Quality of Life (WHOQoL) Bref assessment tool; physical, psychological, social, and environmental health with the overall focus on evaluating quality of life. The data was analysed thematically using QSR NVivo 11.

**Results:**

Of the 37 participants, majority (54%) of them were females, and a considerable proportion (94%) had attained formal education. The most prolonged duration of persons living with HIV among the respondents was 25 years. Four main themes were identified: physical health, social well being, environmental health, and psychological health. The study further revealed that EPLHIV reflect on their condition, face the dilemma of disclosing their status, are confronted with inadequate privacy for health care, wait long in the queue at the health facilities to be attended to, and have no focused or tailored HIV services for the EPLHIV and experiences worsened physical conditions.

**Conclusions:**

The study’s findings shed light on the quality of life among EPLHIV, offering new evidence. Consequently, some of the conditions related to the various domains such as stigma, delay in receiving health care could negatively influence the QoL of EPLHIV. To improve the QoL of EPLHIV, it is imperative to implement specialized services tailored to the unique needs of EPLHIV. Additionally, healthcare workers at ART clinics should receive training in psychological and geriatric care counseling, considering the increasing life expectancy of EPLHIV. There should be counseling sessions on disclosure and non-disclosure to help those who want to disclose their status do so.

## Introduction

Globally, HIV continues to be a major public health concern [1,2]. It is estimated that about 38.4 million people were living with HIV and 1.5 million new infections in 2021 [3]. Sub-Saharan Africa (SSA) accounted for the highest burden, home to about 70% of the estimated population and about 47% of the reported new infections [1,3]. The development and rapid scale-up of ART has contributed to a significant reduction in HIV-associated morbidities and mortalities, resulting in an increasing life expectancy [1,4]. Antiretroviral treatment has transformed HIV from a death sentence to a chronic condition which can be managed for a long time, leading to an elderly HIV population requiring lifelong healthcare needs [5,6]. The proportion of all people living with HIV (PLHIV) who are 50 years and above increased from 8% in 2000 to 16% in 2016 [7].

In Ghana as of December 2023, an estimated 334,095 people were living with HIV of which 40% are aged 50yrs and above. In the same year, 151,520 of the estimated PLHIV were receiving ART and 34% of these individuals were ≥50 years a significant increase from the 19% reported in 2020 [8].

Despite the increased survival rates of HIV patients, the psychological, social and other consequences which contribute to their general well-being is being compromised [9]. Ageing with HIV condition presents several challenges which affect their general well-being and quality of life. These challenges are related to social, psychosocial, physical and their physical environment [10]. Elderly PLHIV suffers from stigma, depression and mental health conditions [11]. Lack of physical activities, poor socio-economic status, excessive intake of alcohol and poor dietary uptake could aggravate and worsen conditions of older people [7,12].

In addition, evidence shows that PLHIV develop non-HIV health conditions than those without the condition [13]. The situation is more difficult with the older population as ageing is associated with the double burden of diseases [4,14]. Most EPLHIV are at risk of experiencing non-communicable diseases such as hypertension, diabetes, kidney disease and others. The HIV condition and non-communicable diseases could lead to co-morbidity, thereby affecting the QoL of the elderly. The QoL for PLHIV, especially the elderly (50 years and above), who are already vulnerable in most Lower and Middle-Income Countries (LMICs), including Ghana, has been neglected [9] which affects their general well-being.

Despite the significant improvement of ART to improve the well-being of people living with HIV and the complex challenges EPLHIV are confronted with, there are few studies examining the QoL of the elderly persons living with HIV in SSA and especially in Ghana [12,15,16].

The few studies on the quality of life among the elderly show mixed results. While some studies have reported poorer QoL [17,18], others have reported good QoL [19]. In Ghana, few studies have examined the QoL among PLHIV, and these studies focused on the general population [15,20–23] but not the elderly population. Hence, there is a paucity of data on the QoL among the EPLHIV. Conducting a study on the quality of life among EPLHIV is essential to address the unique interplay of age-related health challenges, psychosocial stressors, and healthcare disparities, thereby informing targeted interventions and policies. Therefore, this study sought to examine the QoL of the elderly population using the qualitative approach to provide evidence to help improve their well being.

## Materials and methods

### Study design

This study utilized a cross-sectional, qualitative methodology using FGDs and KIIs to examine the quality of life among elderly persons living with HIV. Semi-structured interview guides were used to conduct Key Informant Interviews with stakeholders and Focus Group Discussions with EPLHIV, allowing for in-depth exploration of the subject matter.

### Study settings

The study was conducted in the Greater Accra region of Ghana, located within the country’s coastal part. The region is one of the 16 administrative regions in the country and one of the smallest in terms of landmass. It is the most populated region, with a population of 5,446,237, accounting for 17.7% of Ghana’s total population [24]. According to the 2022 Ghana HIV estimates report, the Greater Accra region is the region with the highest number of PLHIV, accounting for 85,447 (24.1%) of the estimated number of people living with HIV in the country Within the same year, it recorded a prevalence of (2.05%) among adults (15–49) years, exceeding the national prevalence of 1.66% [25]. Again, of all PLHIV and accessing ART services in the region, 34% are 50 years and above [25].

### Study sites

The study sites selected for the FGDs were the Tema General and Achimota Hospitals. These facilities were purposively selected because they are among the facilities with the highest numbers of EPLHIV in the region. [26]. These facilities also provide comprehensive service for persons living with HIV.

### Participants

The participants for this study were Elderly Persons Living with HIV and Key informants. In this study, we defined an elderly person as anyone who is 50 years and above [7,9,10]. Key informants included Service providers at the ART clinics, Officers at the National AIDS/STI Control Programme (NACP), HIV programme coordinators, Leadership of the National Association of Persons Living with HIV (NAP+) and other implementers of HIV prevention programmes in the country. Twenty-nine (29) elderly persons living with HIV and receiving care at the two health facilities were selected for the FGDs while eight (8) key informants were selected for the KIIs.

### Selection of participants

The study participants were selected in two phases using stratified purposive sampling method. In the first phase, EPLHIV were purposively selected from two health facilities where they receive health care for the FGDs. This ensured that the participants had first-hand experience with HIV and its impact on their lives. The health workers assisted in the selection of participants. In the second phase, Key Informants were purposively selected based on their expertise and experiences in providing services to EPLHIV at the facility level, as well as their involvement in the implementation of HIV interventions at the national and sub-national levels. Again, representatives of social network groups for PLHIV were included as key informants. These categories of key informants were chosen because they are integral stakeholders in the development and implementation of HIV policies.

### Data collection procedure

Data collection for the study was carried out in April 2023 using a semi-structured interview guide developed and pilot tested at a health facility providing service for HIV patients in Accra. The participants were selected for the study based on the following processes; Permission was sought from the management of the health facility to use the facility for the study. After which, the health workers assisted in recruiting participants for the study. Participation in the study was voluntary, and all participants signed a written informed consent form indicating their willingness and agreement to participate in this research. Permission was sought from all participants to record the interviews and FGDs.

#### Focus group discussions.

Four (4) FGDs were conducted with EPLHIV to explore their perceptions on QoL. The FGDs consisted of two male and two female’ groups with each group comprising 7–8 individuals (N = 29). The sex disaggregation became necessary following feedback from the pretest on the need to separate males from females to enable interactive discussions The participants were arranged in a horseshoe position, with the moderator and note-taker sitting in the centre. All the FGDs were conducted in the local language, with each meeting lasting between 45 and 60 minutes. During the discussions, participants were allowed to express their views on any posed question before moving to the next one. Probing techniques were employed to clarify any ambiguous points or explore emerging areas of interest. The FGDs were conducted at the two ART sites (health facilities). Each discussion had at most eight (8) participants, a moderator and note taker.

All participants were allowed or given the opportunity to answer a question asked before proceeding to the next question. At the end of the FDGs the moderator summarised all key issues which merged for participants to validate as a form of member checking.

#### Key informant interviews.

Key informant interviews were carried out with personnel from the NACP, leadership of PLHIV, implementers of HIV programmes and service providers at the national and sub national level.This was to explore their perceptions on the quality of life of EPLHIV. These participants were selected due to their perceived knowledge of HIV programming and interventions in Ghana. In all, eight (8) KIIs were conducted.

The interviews were conducted one-on-one with the key stakeholders:

Both the KII and FGD interview guide covered the four (4) domains (Physical, Psychological, Social and Environmental health) of the WHO’s quality of life (WHOQoL) Bref assessment tool. All interview sessions were moderated by trained graduate research assistants with expertise in qualitative research.

### Data analysis

Interviews conducted in English and Twi were recorded and transcribed verbatim. A code was developed by reviewing 10% of the transcripts. The lead author coded the data, author PNT reviewed the coding trail to approve the coding. The data were analysed inductively with NVivo QSR software using the six thematic approaches recommended by Braun and Clarke [27].

The data was analysed based on themes, and this involved searching through the transcripts to find a repeated pattern of meaning and organising them into various levels of themes, such as basic, organised, and global themes. First, each transcript was read multiple times to familiarize with the data while noting emerging issues. This was followed by assigning codes to the appropriate segment of the transcripts. The assigned codes were organized to generate cross-cutting themes from all the transcripts. At this stage, different codes from the transcripts were sorted into potential themes, as all relevant codes were collated within the identified themes.

### Ethical approval

The study obtained ethical clearance from the Ghana Health Service Institutional Review Board (GHS-ERC:033/08/22). The clearance was used to obtain permission to undertake the study in the selected health facilities and among the study participants.

## Results

### Characteristics of study participants

The background characteristics of participants for the KIIs and FGDs are shown in Table 1. Thirty-seven (37) participants were recruited for this study, which was determined when global saturation was reached. The participants included Key Informants (n = 8) and FGDs of EPLHIV (n = 29). Many of the study participants were within the age range of 50 years (n = 25), with the oldest participant being 80 years. The majority of participants were females (n = 20), and many of the respondents had attained formal education (n = 35). In addition, most participants were married (n = 17), and a relative number were separated/divorced or widowed (n = 18). Many of the respondents from the FGDs work in the informal sector (n = 22), and the highest number of years living with HIV among respondents was 25 years.

**Table 1:** Background characteristics of participants.

Background characteristics	Key informants interviews	FGD (EPLHIV)	Total
Sex	4	13	17
Male	4	16	20
Female			
**Age**			
30-39	2	0	2
40-49	4	0	4
50-59	1	24	25
60-69	1	4	5
70+	0	1	1
**Educational level**			
No formal education	0	2	2
Primary	0	14	14
Secondary	0	10	10
Tertiary	8	3	11
**Marital status**			
Single	1	1	2
Married	7	10	17
Separated/Divorced	0	9	9
Widowed	0	9	9
**Occupation**			
Unemployed	0	4	4
Informal employment	0	22	22
Formal employment	8	2	10
Retired/Pensioner	0	1	1
**No. of yrs. living with HIV**			
1–3	0	5	5
4–6	0	10	10
7–9	0	1	1
10+		13	13
**Total**	**8**	**29**	**37**

Source: Field Work, (2023).

### Quality of life of EPLHIV

**Fig 1:**
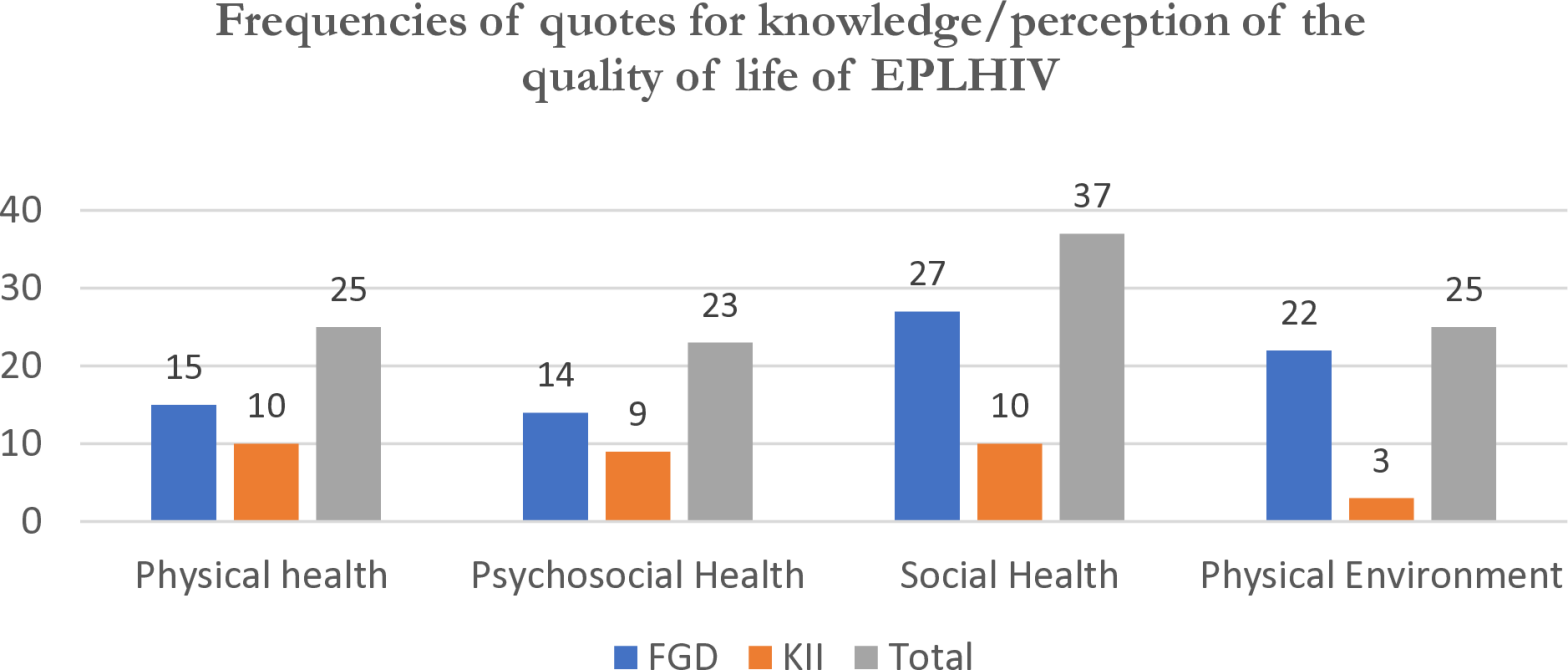
Frequencies of quotes for knowledge/perception of the quality of life of EPLHIV.

Four main domains of the WHO quality of life Bref tool were explored. These are physical, psychological, social, and environmental health. Figure 1 shows the views of participants for each domain of the QoL.

#### Physical health.

Most EPLHIV expressed that they have challenges with their physical health, while few had no challenges. Key Informants indicated that EPLHIV are faced with several physical challenges, such as pains and lack of physical strength.

Both the EPLHIV and Key informants mentioned that EPLHIV feel pains within their body, waist, leg, and knee, which sometimes makes it difficult for them to move and sleep. Due to this, some EPLHIV have resulted in herbal and orthodox medicine to relieve them from their distress.

*“Some of them have issues; these are bone, joint, and bone aches. They will come and complain about it. A percentage of them need aid in that area, but a greater majority of them have one complaint or the other, yes”* **(R1, KII, Male).**

Some EPLHIV indicated that they lack the strength to perform their daily activities. A Key Informant mentioned that most of the EPLHIV do not have the strength to perform their daily activities compared with those who do not have HIV.

*“…For the majority of them…their physical health is not good as compared to their age mates who do not have HIV because the others are also facing the non-communicable diseases that we’ve talked about, that is, hypertension, diabetes and all that, PLHIV are also facing the NCDs, and on top of that, the medicines they are taking for HIV also has some challenges for them”* ***(R2, KII, Male)***.

On the other hand, a few participants expressed that they do not have physical challenges. A participant indicated that he used to have physical challenges, but after receiving treatment, he is relieved from the pain and can now sleep.

*“When I was first diagnosed with HIV, because of how I was thinking and other staff, it made me reduce weight, I could not eat well, and when it was late too, I would not sleep until daybreak before I could have a little sleep but as to when I started the treatment, all the thinking and sleepless night have stopped, and I can now eat”* **(R2, FGD, Male, Facility 1).**

#### Psychological health.

The study further revealed that almost all EPLHIV experience issues relating to their psychological health, while a few had no challenges. Among the psychological challenges include thinking a lot about the HIV condition, getting worried and scared as PLHIV. These psychological issues, in some cases, contribute to an increase in their blood pressure levels.

Some EPLHIV indicated that they started thinking about their HIV condition when they began taking antiretroviral drugs. This continued until they realized that the thinking would not help, so they had to stop. For instance, one EPLHIV indicated that she stopped thinking when she started taking the antiretroviral drugs. In addition, the fear of being seen at the hospital for antiretroviral drugs makes some people think a lot.

*“Firstly, when I contracted the virus, it became a burden to me. I used to think if I was about to die or not, but as the medicine came, I do not think about the sickness anymore as I used to”* (***R2, FGD, Female, Facility 1).***

There were instances where some participants expressed that they get worried about their conditions a lot to the extent that they ask themselves questions about how they contracted the disease. As a way of overcoming this, an EPLHIV mentioned that it was the health workers who advised her to stop worrying about her condition since people were not aware of her illness.

*“When you are told you have something inside you that cannot be removed from you... People get anxious, they are worried, and some of them are very depressed, especially if they don’t have the right support. So, their psychological status is not the same as that of somebody who does not have the disease. So, a lot of them are worried”* ***(R2, KII, Male).”***

Some participants were scared of their condition due to the consequences.

“*At first when I realized I had this sickness, what I knew previously is that when you contract this sickness, it means you are about to die. After two years you will die and a whole lot. This scared me when I saw it on TV”* (***R6, FGD, Female, Facility 1).***

On the other hand, a participant indicated that she does not experience psychological problems because of his secretiveness and adopting a strategy of not thinking about it.

*“It is a problem, because when you have this problem it distances you from your friends, family members too, so you must be very secretive, and you should not be thinking about it because it might lead to other health issues and always feel free to interact with people”* ***(R1, FGD, Female, Facility 1).***

#### Social health.

The findings revealed that many EPLHIVs are unable to disclose their status. Few indicated that they had fully disclosed their status, while others had partially disclosed their status primarily to their immediate family, thus, their partners and children. Most participants have not disclosed their status to others due to the fear of stigma and loneliness.

*“…But there is one thing about this condition, if you have a friend and you tell him or her, that fellow will not come close to you again, because of that I don’t discuss my health issues with anyone, so I move free with them as we were from the beginning because once they get to know your problem, they start to withdraw themselves from you as if you are no more a human being”* ***(R2, FGD, Male, Facility 1)***.

Some EPLHIV expressed that they had partially disclosed their status to some of their family members. Some EPLHIV said that they have a good social relationship with some of their immediate family members who are aware of their status, and some of them even support them in visiting the health facility for treatment and taking medications. A Key Informant indicated that HIV patients who can disclose their status to their family members get more support from them than those who fail to disclose.

*“For my situation, only my wife knows about it, and I am okay with it, and even sometimes I come here with her anytime I come here”* ***(R1, FGD, Male, Facility 2)***.

*“Okay, so, generally, HIV has its associated stigma. So, most people are unable to disclose their status to their partners. For those who can do that and their partners accept it, their social condition is much better than those who did not disclose it to their partners. The condition now serves as a reason for some partners to divorce or separate from them”* ***(R3, KII, Male).***

Few EPLHV had fully disclosed their status to their family members and other people. Consequently, some of them have been neglected, while others are stigmatised. A participant indicated that her family members have distanced themselves from her, and she does things mostly on her own, but their attitude does not bother her. A key informant indicated that EPLHIV should be mentally prepared to face the consequences of disclosing their status because it comes with consequences such as loneliness and stigma.

*“So, a lot of them don’t get the support they need from their family members, friends, and so on because of the stigma associated with the disease. People think that even by coming into contact with them, just sitting, drinking with them, you can get the disease. So, most people do not relate to them well, and in that way, some of them are isolating themselves from the public, and those who don’t have the courage tend even to stigmatize them”* ***(R2, KII, Male).***

Participants who had no challenges with their social health indicated that through the help of the teachings from the health professionals, they have been able to overcome their social challenges and even assist their new colleagues in helping them overcome their challenges.

*“*A*t first, I used to have a problem, but the teaching here and that of what I have also been doing here and the learning and training I have been going through has strengthened me and given me the opportunity to teach the other people that they are now coming here, and motivates them”* **(R4, FGD, Female, Facility 1)**

#### Physical environment.

Concerning the physical environment of EPLHIV, accessing health facilities, training of health workers on stigma, good health care at the health facility, cost of receiving care, unavailability of focus care for the elderly and long hours spent at health facilities getting treatment and care were also identified as some of the themes.

Regarding accessing health facilities, there were issues related to distance and the cost of transportation to the health facility.

*“Some of my colleagues travel long distances to access their ARVs and often face challenges with transportation, leading to difficulties in accessing care. This can have negative consequences on their health. Financial constraints prevent some from renewing their insurance, causing them not to be able to pay for other services”* ***(R8, FGD, Male, Facility 2).***

Some EPLHIV also mentioned the need for training on stigma for some healthcare providers due to how they treat EPLHIV. The training is relevant as stigma could prevent people from receiving care at health facilities.

*“Even after the stigma training with the staff, some staff are not behaving well. If this is happening, we should reorganize the stigma training because we might have received new staff who have not received such training. So that if anyone is coming here, the person does not panic or fear coming here because of such issues”* ***(R4, FGD, Male, Facility 2)***.

A key informant indicated that the physical environment of EPLHIV is enabling EPLHIV to receive good care due to their access to information that is usually provided through counselling sessions that are offered to them at health facilities. Others indicated that the health workers talk to them politely and take very good care of them. This is expressed in a participant’s quote below:

*“With me, I panicked from the beginning when I came here, but with the staff here, the way they pamper us, especially with the madam that takes the blood samples and checks our status, the way she talked to me before she even told me the result, it made me feel some kind of relieve within me together with the doctors and the other staff that works here, they talk with me very well, so since then I also felt like am a human being too and since then I walk free and whenever I want to come here I feel like am coming to my siblings, so to me am okay”* ***(R1, FGD, Male, Facility 2)***.

In addition, many participants expressed that the cost involved in receiving HIV care as EPLHIV is one of their major challenges. These are expressed in the statements below:

*“As for the counselling in the morning, they give us a talk on everything, so I have no problem there. But my problem is the sertraline we buy. Sometimes, when you come, what to use in buying the medicine is difficult; how will you be able to buy the sertraline”* ***(R4, FGD, Female, Facility 1)***.

The unavailability of focused care for elderly people or EPLHIV was also realized in the study. Participants mentioned that there are no specific arrangements for elderly people receiving care in health facilities. Hence, they spend long hours visiting these health facilities, which also affects their health, such as EPLHIV. They are subjected to long stares from people as they wait to receive treatment and care, which makes them feel uncomfortable. For instance, some participants mentioned that:

*“Waking up early to come to this place and staying long here is my challenge. Because if an elderly person like me comes and sits here with the younger ones, we start bickering. If they can give priority to the elderly persons, it will be good, then someone from 50 years and above when she comes won’t have to join the queue or wake up at dawn, that kind of priority would be good”* ***(R1, FGD, Female, Facility 2)***.

Some key informants indicated that the facilities that are available for EPLHIV are not adequate; hence, they are usually not provided with holistic care or treatment. Also, facilities that provide advanced treatment are typically located in the cities; hence, EPLHIV, who live in remote areas, are unable to have access to good treatment and care.

*“The facilities available to take care of them holistically are not adequate. In the care of somebody living with HIV, you need several dimensions and several domains to provide holistic treatment for them. Most of the hospitals for HIV patients are in the regional, district, and national capitals. So, some infected people living in remote areas don’t have access to those facilities. Okay, so the physical environment is not built to support them”* ***(R2, KII, Female)***.

## Discussion

This study aimed to explore the quality of life among elderly persons living with HIV. The findings highlighted the main domains of the WHOQoL assessment, including physical, social, and psychological health and the physical environment. The study’s results show diverse reasons for the quality of life among EPLHIV. Improving the quality of life of EPLHIV is paramount due to the burden of diseases associated with ageing. From this study, the general well-being of EPLHIV was perceived to be poor due to the effects of HIV and other health conditions affecting them. This finding contradicts studies that have reported higher proportion of good quality of life among EPLHIV [11] but is similar to studies that reports moderate general well-being of EPLHIV [2]. The domains contributing to the quality of life of the EPLHIV are discussed below.

With the physical domain, EPLHIV mentioned pains, lack of physical strength, and constant intake of medications (Pill burden) to prevent an increase in blood pressure and other non-communicable diseases as the challenges they face. The key informant’s perception confirmed this as they also emphasized that the physical condition of EPLHIV is not good due to ageing, the double burden of the disease, and the constant intake of medications. The finding of this study corroborates the results of other studies [5,14] that reported on poor physical conditions of EPLHIV. In this study, poor physical conditions are expected due to the age of the participants. Most participants were 50–59 years old, and this age bracket is mainly associated with non-communicable diseases such as diabetes, arthritis, and hypertension [28]. These conditions sometimes predispose people to physical pain. Hence participants in this study were more likely to be exposed to other non-communicable diseases thereby exerting pressure on them to feel pains, not having enough strength, and consequently an increase in blood pressure due to lifestyle activities.

On the physical environment, challenges in accessing health facilities, according to the EPLHIV are due to the cost of transportation and the long distance to the health facilities. These problems are compounded by long hours spent at the health facilities. In this study, the long distance to health facilities for medication is due to the few or selected hospitals that provide services for PLHIV. In Ghana, HIV services are offered at the polyclinics, district, regional, and teaching/tertiary hospitals. For instance, Prevention of Mother-Child Transmission (PMTCT), HIV Testing and counselling, ART patient counselling, and other major services are provided at the district, regional and tertiary hospitals. At the same time, Early Infant Diagnosis (EID) sample collection and testing are done at health centres [29]. This makes it very difficult for persons living in places where there are only Community Health and Planning Services (CHPS compound) and health centres to access all HIV services. They have to travel to places where there is at least a district hospital for HIV comprehensive care services. Knight et al., [30] reported that in South Africa, EPLHIV has difficulty accessing health care due to distance, and this, therefore, reduces the contact hours of PLHIV and health workers. These problems could be reduced if health centres and CHPS compound facilities are selected to provide services to reduce the distance in accessing health care [31].

In addition, there is no tailored or focused health service for the elderly. This sometimes delays EPLHIV in accessing health care. The challenge is that the needs of the elderly are complicated than those of any age group. Waiting for an extended period to be attended to at the health facility could also lead to complications, mainly for those with non-communicable diseases. In terms of policy direction, it is imperative the elderly get their clinic days to enable health workers to attend to them very well.

With regards to the social domain, almost all participants and stakeholders indicated that Elderly Persons Living with HIV are faced with the dilemma of disclosing their status to their family members and friends due to stigma and discrimination. Their social life is impacted by HIV-related discrimination and stigma, which consequently could lead to isolation and fear [4,5]. Vrontaras et al., [5] reported that HIV stigma is a barrier to friends, family, and access to healthcare. Though the disclosure of HIV status would help elderly persons to get the needed social support from family and friends, most of them fear that the outcomes of the disclosure experiences could potentially affect individuals’ acceptance of their HIV status and rather limit their social support leading to rejection and consequently loneliness [5]. Due to the consequences associated with disclosure, most people, therefore, prefer dying with the condition rather than disclosing their status to family and friends [32]. Though there has been education and sensitization on HIV, stigma and discrimination among HIV patients still persist.

The socio-ecological framework is an interdisciplinary approach that considers various factors within an individual’s environment, impacting their health and well-being. The framework identified personal, interpersonal, community and structural factors that influence the well-being of people. In this study, friends and family members play a crucial role in shaping the quality of life for EPLHIV. When individuals disclose their HIV status, they often receive support from their social network, which positively impacts their well-being. Regarding community and structural factors, health workers and the process of receiving care at health facilities significantly influence the quality of life for EPLHIV. Access to quality healthcare services contributes to better outcomes and overall well-being. Hence, understanding and addressing these factors within the socio-ecological context can lead to improved quality of life for individuals living with HIV. It highlights the importance of social support networks, healthcare access, and community resources in enhancing well-being.

### Limitations, strength, policy and practice implications

- This study has some limitations. It was an exploratory study limited to EPLHIV attending HIV clinics in Urban areas in Accra. The sample was limited to those receiving care in urban areas, which is unlikely to reflect the views of EPLHIV receiving care in rural areas.
- In addition, the study was conducted in one region, therefore, it is very relevant to note that the findings are more applicable in the region and cannot be generalised to other parts of the country.
- Also, not all individuals are comfortable discussing their health issues in public. Hence, the use of FGDs among the EPLHIV probably limited people in sharing their experiences due to social desirability, thus the presence of other people due to the nature of HIV/AIDS disease.
- However, FGDs also allow us to understand what the group as a social milieu think. This helped in shaping contrast and similar opinions.
- Despite the limitations, this is the first  study in Ghana examining the quality of life among EPLHIV.
- The findings underscore the need for the incorporation of tailored HIV services for them as part of regular Inservice training for HIV service providers.
- The study findings will also help policymakers adopt strategies to help improve the living conditions of EPLHIV by revising the HIV policy to include specific interventions targeting the quality of life of EPLHIV in Ghana.

## Conclusion

The findings of the study provide evidence on the quality of life among the elderly. This includes the negative and positive experiences related to quality of life. The study revealed that most EPLHIV have physical, social, environmental and psychological problems. It is, therefore, very important that we get tailored clinic days for Elderly People Living with HIV for their needs to be addressed. Again, there should be gerontology health workers trained in HIV counselling to help EPLHIV with problems on their health.

## Supporting information

S1 Table Thematic table. (DOCX)

## Abbreviations:

EPLHIV: Elderly Persons Living with Human Immunodeficiency Virus
FGD: Focus Group Discussions
HIV: Human Immunodeficiency Virus
KIIs: Key Informant Interviews
QoL: Quality of Life
ART: Antiretroviral Treatment
SSA: sub-Saharan Africa
NACP: National AIDS/STI Control Programme
WHOQoL: World Health Organisation Quality of Life.
