# Supplementary material for: Exploring quality of life among elderly persons living with HIV in Accra, Ghana
Source: PLoS One. 2025 Jun 13;20(6):e0324824. doi: 10.1371/journal.pone.0324824 (PMC12165405; doi:10.1371/journal.pone.0324824)
Supplement: S1 Table — (DOCX) [file pone.0324824.s001.docx]

Thematic table

| **Main theme** | **Sub theme** | **Quotes** |
| --- | --- | --- |
| Physical health | Body Pains | For me, it's my waist. Because of that, sometimes my legs become stiff, and I can't walk fast” ***(R6, FGD, Female, Facility 1).***  “Some of them have issues; these are bone, joint, and bone aches. They will come and complain about it. A percentage of them need aid in that area, but a greater majority of them have one complaint or the other, yes” (R1, KII, Male). |
|  | Lack of strength to perform daily activities | *“…For the majority of them…their physical health is not good as compared to their age mates who do not have HIV because the others are also facing the non-communicable diseases that we've talked about, that is, hypertension, diabetes and all that, PLHIV are also facing the NCDs, and on top of that, the medicines they are taking for HIV also has some challenges for them”* ***(R2, KII, Male)****.* |
|  | Relieved from the pain | *“When I was first diagnosed with HIV, because of how I was thinking and other staff, it made me reduce weight, I could not eat well, and when it was late too, I would not sleep until daybreak before I could have a little sleep but as to when I started the treatment, all the thinking and sleepless night have stopped, and I can now eat"* **(R2, FGD, Male, Facility 1).** |
| Psychological health | Thinking | *“Firstly, when I contracted the virus, it became a burden to me. I used to think if I was about to die or not, but as the medicine came, I do not think about the sickness anymore as I used to”* (***R2, FGD, Female, Facility 1).***  *“…I can see that there is a rise in my BP compared to that of what I checked at home because of the pressure here and the people I have been surrounded with. Someone might know you from somewhere and make fun of you seeing you here or spreading the information to others seeing you here. Those things also cause psychological effects on us”* ***(R1, FGD, Male, Facility 2).*** |
|  | Worried | *“For me, nobody knows, and I don't know where I got this, but when I came to the hospital, they advised me to stop worrying and my area also no one is aware”* (***R3, FGD, Female, Facility 1).***  *“When you are told you have something inside you that cannot be removed from you... People get anxious, they are worried, and some of them are very depressed, especially if they don't have the right support. So, their psychological status is not the same as that of somebody who does not have the disease. So, a lot of them are worried”* ***(R2, KII, Male)”*** |
|  | Scared | “*At first when I realized I had this sickness, what I knew previously is that when you contract this sickness, it means you are about to die. After two years you will die and a whole lot. This scared me when I saw it on TV”* (***R6, FGD, Female, Facility 1).*** |
|  | No problems | *“It is a problem, because when you have this problem it distances you from your friends, family members too, so you must be very secretive, and you should not be thinking about it because it might lead to other health issues and always feel free to interact with people”* ***(R1, FGD, Female, Facility 1)*** |
|  |  |  |
| Social health | Non-disclosure | *“With me, I have really kept it a secret because people rarely keep secrets. Even with our children, they might forget and disclose it to someone, and there it goes. It becomes a rumour in your vicinity. So, keeping it a secret is perfect, and I have to free my mind from any personal hunt from within”* ***(R2, FGD, Male, Facility 2).***  *“…But there is one thing about this condition, if you have a friend and you tell him or her, that fellow will not come close to you again, because of that I don’t discuss my health issues with anyone, so I move free with them as we were from the beginning because once they get to know your problem, they start to withdraw themselves from you as if you are no more a human being"* ***(R2, FGD, Male, Facility 1)****.* |
|  | Partial Disclosure | *“For my situation, only my wife knows about it, and I am okay with it, and even sometimes I come here with her anytime I come here”* ***(R1, FGD, Male, Facility 2)****.*  *“Okay, so, generally, HIV has its associated stigma. So, most people are unable to disclose their status to their partners. For those who can do that and their partners accept it, their social condition is much better than those who did not disclose it to their partners. The condition now serves as a reason for some partners to divorce or separate from them”* ***(R3, KII, Male).*** |
|  | Full disclosure | *“I am not bothered. When they were not aware of the sickness, we ate in the same plate, but when they became aware of my sickness, they made my plate and bed different. They don’t sleep with me at night any more, and I am not bothered, but through Christ’s death, God will heal me”* ***(R3, FGD, Female, Facility 2).***  *“So, a lot of them don't get the support they need from their family members, friends, and so on because of the stigma associated with the disease. People think that even by coming into contact with them, just sitting, drinking with them, you can get the disease. So, most people do not relate to them well, and in that way, some of them are isolating themselves from the public, and those who don't have the courage tend even to stigmatize them”* (R2, KII, Male). |
|  | No challenge | “A*t first, I used to have a problem, but the teaching here and that of what I have also been doing here and the learning and training I have been going through has strengthened me and given me the opportunity to teach the other people that they are now coming here, and motivates them* **(R4, FGD, Female, Facility 1)** |
|  |  |  |
| Physical environment | Distance and cost of transportation to health facility | *“Some of my colleagues travel long distances to access their ARVs and often face challenges with transportation, leading to difficulties in accessing care. This can have negative consequences on their health. Financial constraints prevent some from renewing their insurance, causing them not to be able to pay for other services.”* ***(R8, FGD, Male, Facility 2).*** |
|  | Training of health workers | *“Even after the stigma training with the staff, some staff are not behaving well. If this is happening, we should reorganize the stigma training because we might have received new staff who have not received such training. So that if anyone is coming here, the person does not panic or fear coming here because of such issues”* ***(R4, FGD, Male, Facility 2)****.*  “*Due to stigma, many people will not even like to go to a facility that is close to them. So, it all bounces back to stigma stigma stigma. And you know, as a person living with HIV, what disturbs me a lot is when you have self-stigma. If you don't deal with self-stigma, it is very difficult to open up or access some services you are supposed to receive. And we encouraged them to pick up their drugs at the pharmacy as NACP programmed. But they will tell you, "I don't want this place. I want this same place. I don’t want anyone to know about my condition." So, you realize it all bounces back to stigma. So, the difficulty is there, but those who are doing well are the people whose families are aware and are supporting them”* ***(R6, KII, Female)****.* |
|  | Good treatment by health care workers | “*For me, I will say that most people accessing care at our facility are okay because, in our daily counselling, we mention these things to them and encourage them to interact amongst themselves. Because we keep telling them where you are now somebody was there years back and has passed there so whenever you doubt, whenever you need clarification try and approach us and we will link you to somebody who will help”* ***(R4, KII, Female)****.*  *“With me, I panicked from the beginning when I came here, but with the staff here, the way they pamper us, especially with the madam that takes the blood samples and checks our status, the way she talked to me before she even told me the result, it made me feel some kind of relieve within me together with the doctors and the other staff that works here, they talk with me very well, so since then I also felt like am a human being too and since then I walk free and whenever I want to come here I feel like am coming to my siblings, so to me am okay”* ***(R1, FGD, Male, Facility 2)****.* |
|  | Cost involved in receiving HIV care | *“As for the counselling in the morning, they give us a talk on everything, so I have no problem there. But my problem is the sertraline we buy. Sometimes, when you come, what to use in buying the medicine is difficult; how will you be able to buy the sertraline.”* ***(R4, FGD, Female, Facility 1)****.* |
|  | Non-availability of focused care for the elderly | *“Waking up early to come to this place and staying long here is my challenge. Because if an elderly person like me comes and sits here with the younger ones, we start bickering. If they can give priority to the elderly persons, it will be good, then someone from 50 years and above when she comes won’t have to join the queue or wake up at dawn, that kind of priority would be good”* ***(R1, FGD, Female, Facility 2)****.*  *“And also, where do we sit when we come to collect our medicines? I'll plead that you look for another place. If you sit there and someone you know is passing by, you must turn away because people talk. Some people sit at where the AIDS patients sit but are not aware of the way they will carry it around. I plead that you give us another place”* ***(R3, FGD, Female, Facility 1)****.* |
|  | Inadequate facilities | *The facilities available to take care of them holistically are not adequate. In the care of somebody living with HIV, you need several dimensions and several domains to provide holistic treatment for them. Most of the hospitals for HIV patients are in the regional, district, and national capitals. So, some infected people living in remote areas don't have access to those facilities. Okay, so the physical environment is not built to support them”* ***(R2, KII, Female)****.*  *“Even when we come, where we sit to collect our medicines is a problem. Sometimes, some people stare at us when we come for our check-up dates”* ***(R5, FGD, Female, Facility 1)****.* |
|  |  |  |
